# Rectal Cancer: Redox State of Venous Blood and Tissues of Blood Vessels from Electron Paramagnetic Resonance and Its Correlation with the Five-Year Survival

**DOI:** 10.1155/2018/4848652

**Published:** 2018-08-13

**Authors:** A. P. Burlaka, A. V. Vovk, A. A. Burlaka, M. R. Gafurov, K. B. Iskhakova, S. N. Lukin

**Affiliations:** ^1^R.E. Kavetsky institute of Experimental Pathology, Oncology and Radiobiology NAS of Ukraine, Kyiv, Ukraine; ^2^Ukrainian National Cancer Institute, Kyiv, Ukraine; ^3^Kazan Federal University, Kazan, Russia; ^4^V. E. Lashkaryov Institute of Semiconductor Physics NAS of Ukraine, Kyiv, Ukraine

## Abstract

A role of pro- and antioxidants for reducing rectal cancer (RC) incidence in operative, preoperative, and postoperative treatments is still disputable and controversial. The redox state of venous blood and tissues of blood vessels of 60 patients with RC (T_2-4_N_0-2_M_0_G_2_) and 20 donors is studied by means of the conventional and spin-trapping electron paramagnetic resonance (EPR). The intensity of the signals from ceruloplasmin (CP), transferrin (TF), and labile iron pool (LIP) at temperature T = 77 K as well as superoxide generation rate and nitric oxide (NO) levels at T = 300 K is measured. The reduced CP and TF activity and decreased NO levels increased LIP levels and superoxide-generating rates are detected in blood species. Correlation analysis for the five-year survival rate as a function of the extracted values is done. The results show that the intensities of the corresponding EPR signals from the “native” and “trapped” paramagnetic centers can be potentially used for the understanding of the molecular mechanisms underlying the RC progression and treatment.

## 1. Introduction

Biological electron transfer (ET) is crucial for the running of the energy processes of the cell. ET is responsible for the growth and apoptosis of cells and, therefore, can play an important role in the progress of the pathological processes. This transfer is mediated by chains of protein-bound redox (reduction-oxidation) units. The basic redox units of blood are ceruloplasmin (CP), transferrin (TF), “free” iron (also known as labile iron pool, LIP), NADPH oxidase, and iNOS of neutrophils and platelets [1]. The activity of the mentioned units can be investigated with electron paramagnetic resonance (EPR, also abbreviated as ESR for electron spin resonance) techniques. The fundamentals of using the EPR techniques for studying organs and tissues of humans and animals in the norm and pathology were established and developed in 1960-80s [2–10]. It seems that this field is experiencing its second birth in recent years, including numerous applications in cancer related research [11–21].

Various aspects of the EPR utilization in the tumor associated studies with the corresponding references and explanations of the experimental/technical details are given in our publications [11, 13] and additionally for the blood investigations in [22]. From the last one it could be erroneously concluded that EPR of “native” (intrinsic) paramagnetic centers is not a sensitive tool for investigation of blood redox state under the pathological conditions. From other side [23] EPR of 16-doxyl stearic acid as spin probe to measure conformational changes in albumin in blood samples clearly demonstrates its diagnostic utility in patients with cancer including the colorectal cancer (CRC) which comprises rectal cancer (RC) and colon cancer [24]. Changes of intensities of EPR signals in the whole blood corresponding to CP and TF of breast cancer patients under the influence of radiation therapy are pictured in [25].

Paramagnetic centers in human tissues include primarily the molecular complexes containing iron Fe^3+^ (supplied mainly in transferrin and haemoglobin), copper Cu^2+^ ions (ceruloplasmin), and “free” radicals. Human ceruloplasmin is a glycoprotein present in the blood plasma (about 300 *μ*g/ml in healthy adult people) with a molecular weight of 132 kDa. CP in EPR spectra of tissues is usually detected as signal from Cu^2+^ ions with the spectral line at g = 2.05. Ceruloplasmin is implicated in iron metabolism by catalyzing oxidation of Fe^2+^ and thus facilitating the incorporation of Fe^3+^ into apotransferrin. CP oxidizes four Fe^2+^ ions and is involved in four-electron transfer to oxygen, thus hindering nonenzymatic oxidation of iron producing “free” radicals [26]. Apotransferrin is EPR-silent, but when it bounds Fe^3+^ ions one can observe a distinct EPR signal with g ≈ 4.3 from the paramagnetic Fe^3+^ ions due to the high-spin (electronic spin* S *= 5/2) iron in TF.

About (3–4) grams of iron is distributed among body compartments. In normal subjects it is all protein bound. About 70 % of the total iron is circulating, largely in erythrocyte haemoglobin. Up to 25 % of iron is stored in cells in the cytosol as ferritin or in the lysosomes as haemosiderin. Only about 0.1 % of the total body iron is circulating in plasma, all bound to TF. “Free” iron is toxic for blood and tissues [26]. In many pathological processes, including the tumor growth, the amount of “free” iron in blood and tissues increases due to the decompartmentalization of Fe ions from the ferritin and destruction of other heme and nonheme proteins. EPR spectra of “free” iron (labile iron pool, LIP) are detected as a broad signal in the vicinity of g = (2.2 – 2.4) [9, 14, 15].

Comparing with the normal counterparts, cancer cells generate more “free” radicals. When “free” radicals are produced in excessive and uncontrollable amounts, they and their derivative products may react with various cellular macromolecules, such as lipids, proteins, and DNA and may modulate gene expression [27, 28]. Redox state and ET disturbances are associated with the synthesis of reactive oxygen species (ROS) and degradation of matrix proteins, with consequent effects on cell survival, invasion, and metastasis [29]. Matrix metalloproteinases (MMPs) are capable of decomposing extracellular matrix proteins, enhancing invasion of cancer cells. Generally, it is assumed that the synthesis and/or activation of MMP is increased by oxidative stress such as that created by activated neutrophils and ROS, for example [29, 30].

Recent data suggest more important and significant roles for neutrophils and platelets in tumor biology. Neutrophil-to-lymphocyte and platelet-to-lymphocyte ratios have been proposed as independent markers of poor prognosis in patients with cancer, including CRC [31]. A role of neutrophils is controversial. At present it is assumed that there are two neutrophil phenotypes, the so-called antitumour N1 neutrophils and protumorigenic N2 neutrophils. The N1 neutrophils show a direct antitumour effect induced by ROS production as well as antibody-dependent cellular cytotoxicity. Meanwhile, N2 neutrophils are thought to facilitate cancer development via reconstruction of the extracellular matrix, acceleration of angiogenesis and lymphangiogenesis, and immune modulation through protumorigenic cytokine production [32, 33].

In this study, we report the measurements of the redox state of venous blood (also the extracted neutrophils and platelets) of patients with rectal cancer which include EPR of the native paramagnetic centers at liquid nitrogen temperature of T = 77 K and spin-trapping EPR of the reactive oxygen/nitrogen species- (ROS/RNS-) superoxide (${O_{2}}^{\underline{•}}$) and nitric oxide (NO) radicals at room temperature (RT) to exhibit the features of the RC redox state in blood and to demonstrate the EPR capabilities for RC research. The results are compared with those obtained on the pieces of the blood vessels from tumor and the superior rectal arteries. It is a continuation of our many faceted works with some preliminary results presented in [34, 35].

## 2. Materials and Methods

Venous blood of 60 patients who stayed at the Ukrainian National Cancer Institute for treatment (34 men and 26 women, mean age 61 ± 2.3 years) with stage II/III (*Т*_2-4_N_0-2_M_0_G_2_ according to the Seventh Edition of the American Joint Committee of Cancer classification [36], where T factor is the degree of wall penetration of the primary tumor; N factor is the status of lymph node metastasis; M factor shows the presence of distant metastasis; and G describes the grade of the cancer) of adenocarcinoma of the rectum was studied. Diagnoses, stage of disease, and presence of metastasis were established according to requirements of the evidence-based medicine (morphologically, in course of corresponding clinical-instrumental checkup). No other diseases were diagnosed within the investigated group. All participants expressed their prior written consent to take part in the research. All procedures followed were in accordance with the ethical standards of the responsible committee on human experimentation (institutional and national) and with the Helsinki Declaration of 1964 and later amendments. Results for the group of 20 practically healthy people (9 men, 11 women at age of 56 ± 4.1 years) served as control values.

0.5 mL from 6 mL of the collected venous blood sample from elbow vein was poured into the test tube with 0.1 mL of anticoagulant Trilon B solution (3%), then frozen, and stored in a special mold in liquid nitrogen for the estimation of CP, TF, and “free” iron levels by EPR at T = 77 K. The rest (5.5 ml) was used for the determination of NO level and generation rate of ${O_{2}}^{\underline{•}}$ radicals in the whole blood, neutrophils, and platelets at RT by using Fe/diethyldithiocarbamate (Fe/DETC) and 1-hydroxy-2,2,6,6-tetramethyl-4-oxo-piperidine hydrochloride (TEMPONE-H) from Sigma-Aldrich as spin traps. Neutrophils were isolated in a double density (*ρ* = 1.077 g /cm^3^ and *ρ* = 1.119 g /cm^3^) gradient of Ficoll–Verographin at + 4°C by centrifugation at 400g during 45 min [37]. Platelets were extracted after a series of blood centrifugations at + 4°C according to procedure described in [38]. EPR measurements were done by using RE-1307 (USSR, Russia) and Bruker ESP-300 (Bruker, Germany) EPR spectrometers (the spectrometers operate at microwave frequency of 9.5 GHz) at room and liquid nitrogen temperatures (T = 77K). Technical details of EPR measurements and sample preparations with spin traps are given elsewhere [11, 13, 15].

Additionally, to follow the changes in the blood circulatory system connected with adenocarcinoma, samples of the superior rectal artery and blood vessels of tumor tissues from 21 RC patients (12 men, 9 women) and 9 peptic ulcer patients (5 men and 4 women, mean age 59.1 ± 2.8 years) obtained during the surgical interference were frozen, stored in a special mold in liquid nitrogen, and investigated.

Statistical analyses were done using GraphPad Prism 6 and Origin 7.5 programs. Difference between the parameters was considered to be reliable for p < 0.05.

## 3. Results and Discussion 

**Figure 1:**
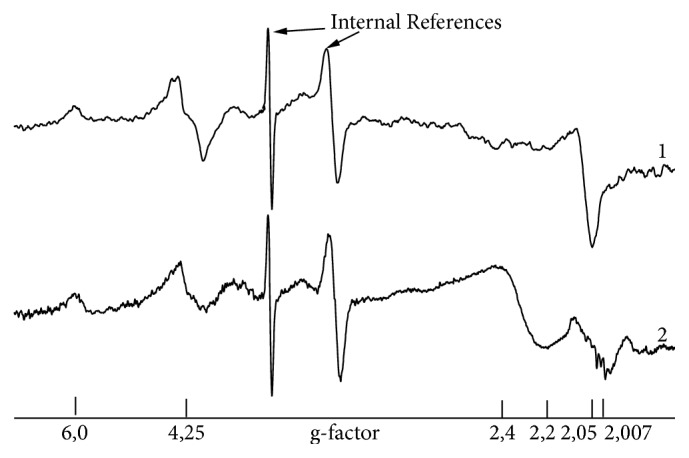
Typical EPR spectra of samples of blood of (1) donor and (2) patient with RC at T = 77 K in the units of g-factor. Description and assignment of the obtained EPR features are given in the text. The internal references signals are from the ruby crystals on the wall of EPR cavity for the quantitative analysis of EPR spectra.

Figure 1 shows the typical EPR spectra of blood samples of donor and patient with RC at liquid nitrogen temperature. To exclude the influence of the resonance frequency onto the shift of the EPR spectra, it is quite common to recalculate and present the spectra in the units of the spectroscopic* g-*factor rather than magnetic field strength following the simple relation

(1) $$\begin{array}{c} h\nu =g\beta B, \end{array}$$

where h is Planck constant, *ν* is a microwave frequency of EPR, *β* is Bohr constant, and B is a magnetic field strength.

There are lines with g = 2.05, g = 4.25 in spectra 1 and 2 in Figure 1; their intensities correspond to CP activity and TF content, correspondingly. Additionally, signals due to methemoglobin (g = 6.3) and “free” iron (LIP, g=2.2 – 2.4) could be detected.

**Figure 2:**
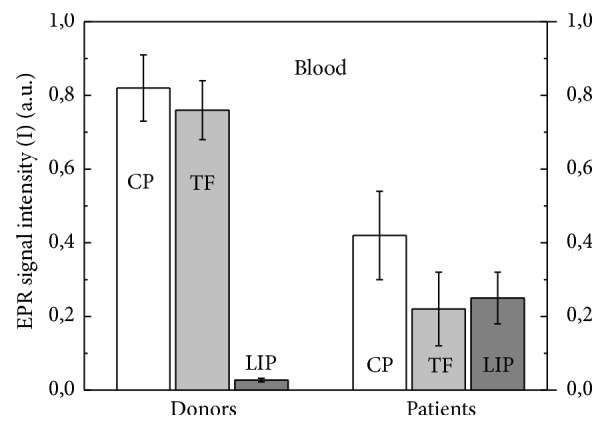
Relative intensities (I) of EPR signals corresponding to CP, TF, and LIP in blood of donors (n = 20, 0.82 ± 0.09, 0.76 ± 0.08, and 0.027 ± 0.010, correspondingly) and RC patients (n = 60, 0.42 ± 0.12, 0.22 ± 0.10, and 0.25 ± 0.070, correspondingly). Data are presented as mean ± SEM.

The results of comparison of EPR intensities for CP, TF, and LIP for donors and RC patients are shown in Figure 2. It follows that CP and TF levels for blood of RC patients are 2 and 3 times less than those for donors, respectively, while growth of LIP level in 10 times is observed (p<0.05).

**Figure 3:**
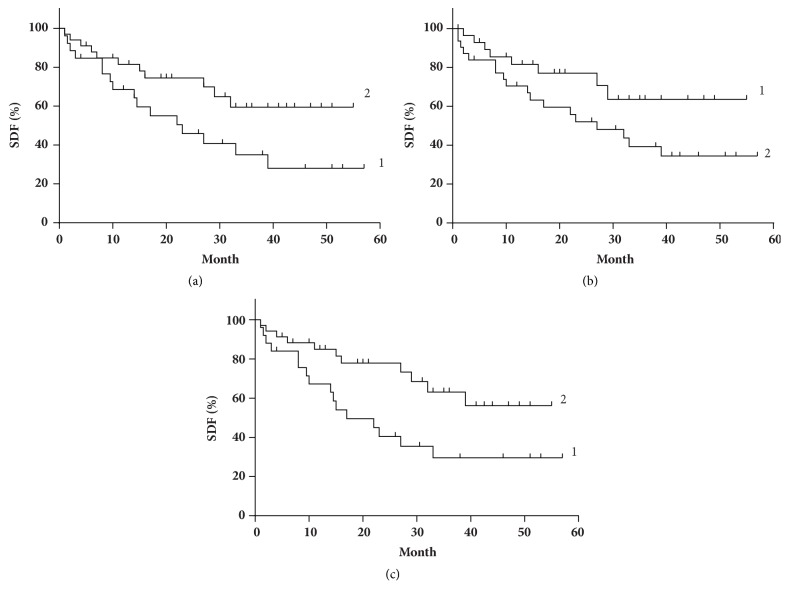
Survival distribution functions (SDF) depending on (a) CP activity in blood for I_CP_ < 0.42 a.u. (curve 1) and I_CP_ > 0.42 a.u. (curve 2); (b) TF level in blood for I_TF_≥ 0.22 a.u. (curve 1) and I_TF_ < 0.22 a.u. (curve 2); (c) LIP level for I_LIP_ < 0.23 a.u. (curve 1) and I_LIP_ ≥ 0.23 (curve 2).

Kaplan–Meier analysis was performed to determine and to compare 5-year survival associated with the CP, TF, and LIP activity/level (Figure 3). The five-year overall survival (OS) of patients with the activity of CP I_CP_≥ 0.42 a.u. (n = 34) was 60%, median survival (SM) was not reached. For the group with I_CP_ < 0.42 a.u. (n = 26) OS = 28%, the median time was of 23 months (*χ*^2^ = 4.08, p = 0.044, Figure 3(a)). For I_TF_≥ 0.22 (n = 29), SM was not reached, OS = 60%. For I_TF_ < 0.22 (n = 31), the median time was 27 months (*χ*^2^ = 3.16, p = 0.076, Figure 3(b)). For the subgroup with I_LIP_ < 0.23 a.u. (n = 33) OS = 58%, SM was not reached; with I_LIP_≥ 0.23 (n = 27) OS = 30%, the median time was 17 months (*χ*^2^ = 4.96, p = 0.026, Figure 3(c)).

Data for the generation rates of superoxide radicals by NADPH oxidase and NO radicals by iNOS of blood neutrophils and platelets are gathered in Table 1. From those, it can be seen that ${O_{2}}^{\underline{•}}$ generation rate of platelets is of 8-14 times higher than in the control species (р < 0.001).

**Figure 4:**
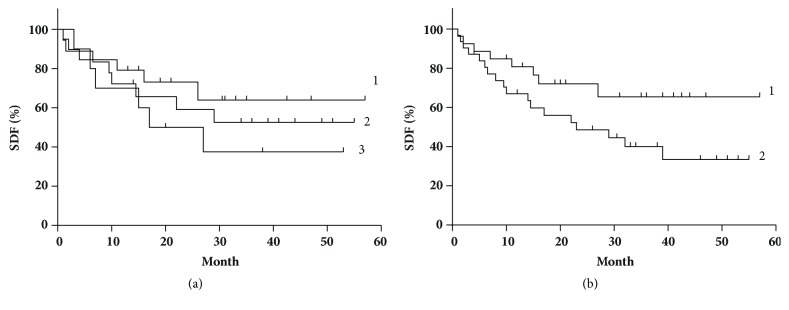
Survival distribution functions (SDF) depending on (a) superoxide-generating activity of NADP·N-oxidase of platelets: 1 –< 2,35 nM/10^5^ cells·min; 2 –(2.5 – 3.0) nM/10^5^ cells·min; 3 - > 3.20 nM/10^5^ cells·min; p = 0,35; (b) NO-generative activity of platelets 1 – > 0.34 nM/10^5^ cells·min; 2 - ≤ 0.34 nM/10^5^ cells·min.

According to the superoxide generation rate data, the patients may be divided into 3 subgroups with the distinct 5-year survival (Figure 4(a)): (1) with the high activity (> 3.20 nM/10^5^ cells·min, n = 11, mean = 3.45 ± 0.11); (2) with the moderate activity (in the range 2.50 – 3.20, n = 18, mean = 2.75 ± 0.03, p < 0.05); and (3) with the low activity (< 2.5, n= 21, mean = 2.35 ± 0.05, р < 0.05). Five-year OS for subgroup 1 was 39%, SM = 22 months; for subgroups 2 and 3, SM was not reached and OS values are of 56% and 62%, correspondingly (*χ*^2^ = 0.88, p = 0.35).

RC patients have the significantly lower levels of NO production (up 3 times, р < 0.01). For the patients with this value of < 0.41 nM/10^5^ cells·min, OS = 31 %, SM = 23 months while for the values of > 0.41 nM/10^5^ cells·min OS = 66% and SM was not reached (*χ*^2^ = 3.11, р = 0.078).

As concerns NO generation rate of platelets, in the subgroup with the high activity of iNOS (> 0.34 nM/10^5^ cells·min) OS = 64%, SM was not reached, and in the subgroup with the low activity of iNOS (≤ 0.34 nM/10^5^ cells·min) SM = 29 months (p = 0.4, Figure 4(b)).

Data for the superoxide generation rate by neutrophils allow dividing the five-year survival results into two groups: one (30% from 60 patients) with the same values as for the control one and the rest (2) with 1.5-2 times (р < 0.01) higher values. It is in correspondence with the dual role of neutrophils in tumor progression (see the Introduction section, [31–33]). For the statistical analysis we have divided the investigated group into two subgroups: (1) with the ${O_{2}}^{\underline{•}}$ generation rate > 0.31 nM/10^5^ cells·min (n = 28) for which OS = 40% and SM = 39 months were observed; and (2) with the ${O_{2}}^{\underline{•}}$ generation rate < 0.31 nM/10^5^ cells·min (n = 32) for which OS = 78 % and SM was not reached (*χ*^2^ = 4.1, р = 0.043, data are not presented graphically). As concerns nitric oxide, no statistically significant correlation between the neutrophils' NO levels and five-year survival was found (*χ*^2^ = 2.3, р = 0.13).

**Figure 5:**
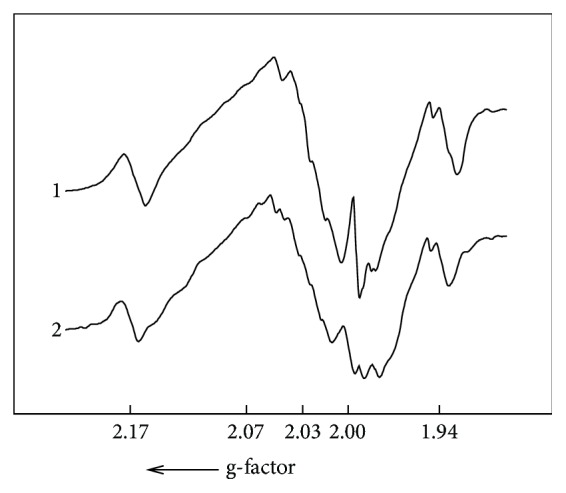
Parts of EPR spectra of blood vessels from rectum tumor (1) and from the superior rectum artery (2) in the vicinity of g-factor ≈ 2.

Significant alterations of the redox state observed in the blood species suggest that the corresponding changes could also be obtained in the tumor tissues and arteries that feed the tumor tissues. EPR spectra from the blood vessels of tumor and vessels from the nearby tissues of RC patients are shown in Figure 5. The main feature of the EPR for RC tissues is a signal with g = 2.03. The intensity of this signal corresponds to the level of NO-FeS protein complexes formed during the interaction of NO• with FeS-proteins of the respiratory chain of mitochondrial membranes, in particular with proteins of N and S clusters in NADPH-ubiquinone reductase and succinate dehydrogenase, respectively [13]. In the vessels of tumors, high levels of NO-FeS-protein complexes (1.8 ± 0.11 a.u., m = 26) are found in comparison with the upper rectal artery (0.58 ± 0.05 a.u.. n = 26) and control group (artery tissues of the peptic ulcer patients, 0.15 ± 0.02 a.u., n = 9) (the nature of other signals presented in Figure 5 is described in detail in [13]).

**Figure 6:**
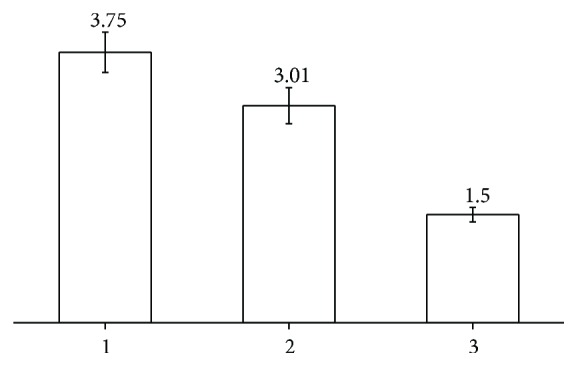
Concentration of NO in vessels from (1) tumor (n = 26, 3.75 ± 0.28); (2) superior rectum artery of RC patients (n = 26, 3.01 ± 0.25); and (3) superior rectum artery from peptic ulcer patients (n = 9, 1.5 ± 0.1). Data are presented as mean ± SEM in the units of nM/g of tissue.

**Figure 7:**
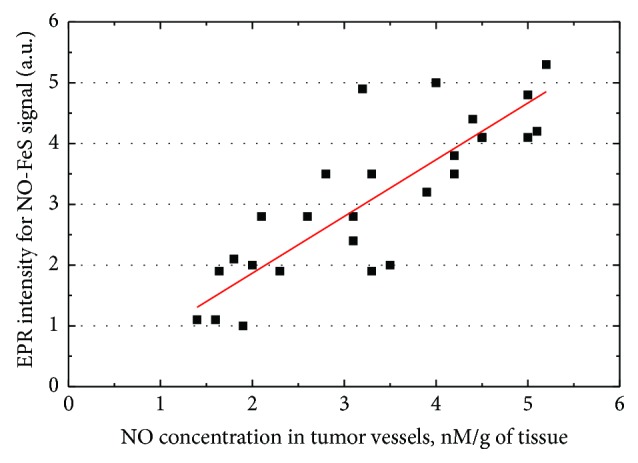
Correlation between the relative intensity of the intrinsic EPR signal at g = 2.03 and NO concentration derived from the spin-trapping measurements in tumor vessels of RC patients (Т3N1М0G2). The straight line is drawn with a slope of 0.93.

We have measured the nitric oxide concentrations in the vessels by the spin-trapping technique. The obtained data are presented in Figures 6 and 7. As follows, in contrast to the blood species, NO levels in the arteries of RC patients are higher than in the peptic ulcer patients' tissues (p < 0.01, Figure 6). Correlation between the NO concentration from the spin-trapping EPR and intensity of EPR signal with g = 2.03 in the tissues of tumor vessels becomes apparent from Figure 7 (r = 0.84, p < 0.05). It shows that the intensity of the intrinsic EPR signal at g = 2.03 can serve (at least in some cases) as a measure of NO amount without applying the expansive spin traps while EPR itself could be exploited as an indicator of pathological changes. No reliable correlation between the NO levels in the investigated tissues and survival rates was established.

As can be seen, the NO production in RC tissues is high and in correspondence with our previous observations for other tumor types [11–13]. Let us now discuss the possible mechanisms which lead to the lowering of the levels of NO and superoxide overproduction in the blood species of RC patients (the feasible ways of changing of the copper and iron related EPR signals are extensively reviewed in the Introduction section). The main generating source for the nitric oxide generation is the inducible nitric oxide synthase (iNOS). NOS enzymes are complex oxido-reductases with the potential to produce ROS as well as, or instead of, NO. Specifically, for NO production, the NOS enzymes require the cofactor tetrahydrobiopterin (BH4). BH4 participates in electron transfer during the two-step oxidation of L-arginine to L-citrulline via an* N-*hydroxyarginine intermediate. In the absence of BH4, NOS enzymes are unable to generate NO by oxidation of L-arginine and become “uncoupled,” whereby reduction of molecular oxygen, driven by electron flow from the NOS reductase domain, can generate superoxide or other ROS [39–42].

## 4. Conclusion

The growth of adenocarcinoma of RC patients (Т2-4N0-2M0G2) is accompanied by the changes in the redox state of blood. Namely, CP activity and TF levels decrease, resulting in appearing of “free” iron and increase of its level. The results show the feasibility of using EPR of the intrinsic paramagnetic centers for the relative simple investigation of blood redox state. Status of the redox-forming parts of blood correlates with the five-year survival rate.

Simultaneously, due to the alteration of NADP·N-oxidase and iNOS superoxide- and NO- generative activities of neutrophils and platelets, the nitric oxide generation activity reduces while the superoxide generation rate grows. Though a relationship between the superoxide generation rate and the five-year survival rates is found, no reliable correlation between the NO levels in the investigated blood species, artery tissues, and survival is established. It emphasizes anew that the link between the NO levels and biological response should be further extensively studied [40].

## Figures and Tables

**Table 1:** Superoxide and NO generation rates in blood platelets and neutrophils of RC patients and control group; NO levels in tumor blood vessels and tissues of adjacent rectum artery.

	${O_{2}}^{\underline{•}}$			NO		
	mean ± SEM	range	mean SEM (control)	mean ± SEM	range	mean ± SEM (control)
Blood, nM/10^5^ cells·min						

Platelets	2.67 ± 0.08	1.75-3.95	0.25±0.01	0.34 ± 0.01	0.25 – 0.49	1.51 ± 0.02

Neutrophils	0.336± 0.010	0.22-0.48	0.230 ± 0.004	0.41 ± 0.11	0.15-0.49	1.45 ± 0.02
n_1_= 18	0.240 ± 0.003					
n_2_= 44	0.36 ± 0.01	0.25-0.48				

## Data Availability

The data used to support the findings of this study are available from the corresponding author upon request.
